# Comparative phylogeographic study of *Hosta sieboldiana* and *Hosta albomarginata* (Asparagaceae) in Japan

**DOI:** 10.1002/ece3.838

**Published:** 2013-10-31

**Authors:** Sangryong Lee, Masayuki Maki

**Affiliations:** Botanical Gardens, Tohoku UniversityKawauchi 12-2, Aoba-ku, Sendai, 980-0862, Japan

**Keywords:** BARRIER, chloroplast DNA, *Hosta albomarginata*, *Hosta sieboldiana*, haplotype diversity, *Hosta*, Japanese archipelago, NJ tree, phylogeography, SAMOVA

## Abstract

We analyzed variations in chloroplast DNA (cpDNA) in the widespread herbaceous species *Hosta sieboldiana* and *Hosta albomarginata* across large portions of their geographic ranges in the Japanese archipelago. Our objective was to compare the phylogeographic histories and phylogeographic structures of the two congeneric species in the Japanese archipelago. The location of the study is Japanese archipelago. We sequenced 1380 bp of noncoding cpDNA from 45 populations of *H. sieboldiana* (*n* = 362) and 55 populations of *H. albomarginata* (*n* = 436) to assess genetic variations within and among populations across almost the entire distributions of the species in Japan. Extant patterns of geographic structure were analyzed using statistical parsimony networks and spatial analysis of molecular variance (SAMOVA). We also used Monmonier's algorithm to detect genetic barriers between regions. Relationships between the populations were examined using a neighbor-joining (NJ) method. Four haplotypes were found for *H. sieboldiana*, whereas eight haplotypes were identified for *H. albomarginata*. Total genetic haplotype diversity (*h*T) and within-population haplotype diversity (*h*S) for *H*. *sieboldiana* were 0.352 and 0.040, respectively, while the values for *H*. *albomarginata* were 0.529 and 0.085, respectively. The population differentiations (*G*ST) for *H. sieboldiana* and *H*. *albomarginata* were 0.839 and 0.886, respectively. The SAMOVA analysis revealed two clusters in *H. sieboldiana* and four clusters in *H. albomarginata*. Differentiations between and among the clusters were supported by the BARRIER analysis and the NJ tree. We detected differences in the population genetic structure between the two species. We found that *H. sieboldiana* had lower haplotype diversity than *H. albomarginata*. These results may be partially explained by the difference in ecological habitats and geographic distributions between the species. *Hosta albomarginata* is more widely distributed than *H*. *sieboldiana* in East Asia including Russia, and this large distribution range would enable more chances to intraspecific gene flow.

## Introduction

Over the past decade, the geographic distributions of intraspecific cpDNA variations have been examined in a variety of plant species to elucidate their postglacial migration history (Petit et al. 2002; Mclachlan et al. 2005; Ikeda et al. 2009; Bai et al. 2010; Kikuchi et al. 2010; Lafontaine et al. 2010; Chou et al. 2011; Higashi et al. 2012; Sede et al. 2012; Cosacov et al. 2013). A principal goal of comparative phylogeography is to infer biogeographic history from recurrent patterns in the geographic distribution of genetic variation in co-distributed species (Bermingham and Moritz 1998; Moritz and Faith 1998; Avise 2000). Comparative phylogeography of woody plants has been especially useful in reconstructing Quaternary forest distributions, particularly in Europe and eastern North America (Palme et al. 2003, 2004; Heuertz et al. 2004; Grivet et al. 2006; Maliouchenko et al. 2007; Saeki et al. 2011), and similar types of studies on Japanese plants have been performed on tree species (Okaura and Harada 2002; Kanno et al. 2004; Okaura et al. 2007; Iwasaki et al. 2012) and alpine plants in temperate zones (Ohi et al. 2003; Fujii and Senni 2006; Ikeda and Setoguchi 2006, 2007; Ikeda et al. 2006, 2008a,b). However, to date, few comparative phylogeographic studies have been conducted for herbaceous species in the Japanese archipelago (Toyama and Yahara 2009).

**Figure 1:**
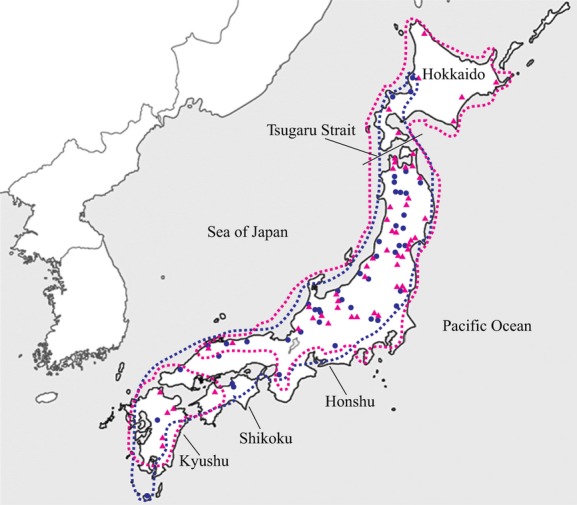
Sampling localities of *Hosta sieboldiana* (blue circle) and *Hosta albomarginata* (pink triangle) in the Japanese archipelago. The dotted lines demonstrate the distributions of *H*. *sieboldiana* (blue) and *H*. *albomarginata* (pink) by Fujita (1976).

The Japanese archipelago was connected to the Eurasian continent by four landbridges as a result of the lower sea level during the last glacial era (18,000–20,000 years ago): Sakhalin, the Kuriles, the Korean peninsula, and the Ryukyu Islands. These connections might have affect on the genetic structures and distributions of many plants (Hotta 1974). Phylogeographic studies on the Japanese archipelago elucidated several genetic disjunct distribution patterns. For example, genetic differentiation exists between populations along the Sea of Japan and the Pacific Sea sides of Japan (Okaura and Harada 2002; Tsumura et al. 2007a,b; Hiraoka and Tomaru 2009; Iwasaki et al. 2012). It might be thought that there are some mountain ranges from northeast to southeast in the Japanese mainland, probably acting as physical barriers to the migration or gene flow of many plant species (Tsukada 1980). The mountain ranges creates a climate boundary, dividing the main Japanese islands into zones with wet winter with heavy snow along the Japan Sea sides and with dry winter with small precipitation along the Pacific sides (Hiraoka and Tomaru 2009). *Fagus crenata* and *Cryptomeria japonica* have shown the genetic divergence between the sides of Japan Sea and the sides of the Pacific sea. Furthermore, a clearly phylogeographic break exists between the northern and central parts and/or western parts of Japan (Tsuda and Ide 2005, 2010; Fujii and Senni 2006; Ikeda et al. 2006, 2008a; Tsumura et al. 2007a,b; Sugahara et al. 2011). The most likely explanation for this phylogeographic pattern might be the multiple colonization events following different glacial episodes with the Japanese archipelago, during repeated glacial and interglacial cycles. Some alpine plants such as *Pedicularis chamissonis* and *Primula cuneifolia* have shown the divergence pattern between the northern parts and central parts. Fujii and Senni (2006) concluded that these divergence times corresponded to the period from the middle of the Pliocene (Tertiary) to the middle of the Pleistocene (Quaternary) in these two species. Some wood plants such as *Chamaecyparis obtuse* and *Aesculus turbinate* have shown the possibility that there have been refugia of high mountains in the western parts. Finally, phylogeographic divergence was detected between Hokkaido and Honshu islands (Aizawa et al. 2007; Hu et al. 2010; Ohsawa et al. 2011). Previous phylogeographic studies of Japanese broad-leaved trees (*Fagus crenata*, Okaura and Harada 2002; *Betula maximowicziana*, Tsuda and Ide 2005; *Quercus mongolica* var. *crispula*, Okaura et al. 2007) generally indicate more recent bottlenecks in the extant populations on Hokkaido than those in Honshu. The populations on Hokkaido probably recolonized from several southern refugia in Honshu during warmer postglacial periods. The genus *Hosta* has probably diversified because of speciation in the archipelago. Fujita (1976) classified 18 species and seven varieties in the genus *Hosta*. The congeneric species, *Hosta sieboldiana* and *H*. *albomarginata* of the *Hosta* genus, are the most common and widespread herbaceous species in the Japanese archipelago (Fig. 1). *Hosta sieboldiana* and *H. albomarginata* are not so close species in the genus *Hosta*. *Hosta sieboldiana* belongs to the section *Helipteroides* although *H. albomarginata* belongs to the section *Nipponosta*. However, the distributions of these two species are similar in Japan, and their overall morphologies and life forms are also similar. So, we think that these two species are good materials for comparative phylogeography. These two species are mostly pollinated by bumblebee species. The flowers of *H*. *sieboldiana* are self-compatible but require pollinators for seed production (Takahashi et al. 1993). Those of *H. albomarginata* are also weakly self-compatible and a facultatively xenogamous species (Suzuki et al. 2002). The seeds with wings of these two species are dispersed by wind. The common pollinator might visit the two species at the overlapping flowering seasons and the hybridization might occur. Although these species sometimes co-occur, they differ in their habitat preferences; *H. sieboldiana* mostly occupies cliffs around waterfalls and steep clines, whereas *H. albomarginata* widely inhabits wetlands. Furthermore, *H*. *sieboldiana* is endemic to Japan, while *H. albomarginata* is distributed in both Japan and Russia (Tamura and Fujita 2013). In addition to the Japanese archipelago, it occurs south of the Amurskaya and Primorsky Territories of the Eurasian continent, Sakhalin, and the Kurils of Russia (Gage et al. 2006).

Because the morphologies of these two species are very diverse, intraspecific taxa have been recognized. Populations of *H*. *sieboldiana* along the Sea of Japan have been treated as *H*. *sieboldiana*, although other populations were regarded as another species, *H*. *montana* (Maekawa 1940). Maekawa (1972) suggested that *H*. *sieboldiana* and *H*. *montana* can be discriminated in the length of peduncle, the color of perianth, the opened angle of the corolla, and the white powder of the back side of leaves. *Hosta albomarginata* has also been regarded as more than one species; the populations from the northern part of the Japanese archipelago have been treated as *H. rectifolia* Nakai, while those from the Kansai areas to the Chugoku areas of the archipelago have been treated as *H. rhodeifolia* F. Maek and the populations in the other regions of the archipelago have been regarded as *H. albomarginata* (Maekawa 1940). Maekawa (1940) insisted that the tube height of the perianth is shorter than the tube width of perianth in the northern populations of *H. albomarginata*. However, these characters could not discriminate between the northern populations and the western populations of *H. albomarginata*. Therefore, Fujita (1976) regarded these three species as one species, *H. albomarginata,* because the range of variations in morphological characters within *H. albomarginata* is not so large. These intraspecies subdivisions might reflect the population genetic structures within these species, which may have been generated by climate oscillations during the last glaciations (Qiu et al. 2009a,b; Lee et al. 2013). Moreover, the inference of different biogeographic histories for the two species has been based on the molecular marker (Albach et al. 2006; Maliouchenko et al. 2007; Toyama and Yahara 2009; Saeki et al. 2011). Therefore, we focused on the geographic pattern of cpDNA sequence variations in the two congeneric species, *H. sieboldiana* and *H. albomarginata*, to infer the phylogeographies of these species and to compare their distribution range histories. This study also serves as an example for elucidating different phylogeographic structures among closely related species differing in ecological habitats.

## Materials and Methods

### Plant materials

Leaf samples were collected from 1 to 10 individuals from each of 45 and 55 populations of *H. sieboldiana* and *H*. *albomarginata*, respectively, to cover the entire distributions of the species in the Japanese archipelago (Fig. 1). In total, 362 individuals and 436 individuals were examined for *H. sieboldiana* and *H*. *albomarginata*, respectively. Both of species rarely propagate vegetatively (but < 1 m^2^ area). We sampled individual plants occurring a few meters apart to prevent the collection of the same genets. Leaf materials were kept in an ultracold refrigerator (−70°C) in Ziplock plastic bags until DNA extractions were carried out.

### DNA extraction and sequencing

Genomic DNA was extracted from the leaves according to the CTAB method of Doyle and Doyle (1987). PCR amplifications were conducted in a total reaction volume of 15 μL containing 10–20 ng of total DNA, each primer at 0.15 μmol/L, 0.1 mmol/L deoxynucleoside triphosphates (dNTPs), 50 mmol/L KCl, 2 mmol/L MgCl_2_, 10 mmol/L Tris–HCl (pH 8.3), and 0.375 units of Taq DNA polymerase (Amplicon Inc., Irvine, California). To examine the geographic distribution of cpDNA in *H. sieboldiana* and *H. albomarginata*, we amplified two noncoding regions (1380 bp) of cpDNA, the *trn*S(GCU)–*trn*G(UCC) spacer and the *trn*L–*rpl*32F spacer, using the primers reported by Hamilton (1999) and Taberlet et al. (1991), which had been successfully amplified and exhibited some variations in preliminary experiments. Double-stranded DNA was amplified after incubation at 94°C for 3 min, followed by 30 cycles of incubation at 94°C for 30 s, 55°C for 30 s (the *trn*L–*rpl*32F intergenic region), 60°C for 60 s (the *trn*S(GCU)–*trn*G(UCC) intergenic region), and 72°C for 30 s, with a final extension at 72°C for 5 min. After amplification, the PCR products were purified using a Gene Clean Kit II (MP Biomedicals, Irvine, California). We sequenced the purified PCR products using a BigDye™ Terminator Cycle Sequencing Ready Reaction Kit (Applied Biosystems, Foster City, California) and analyzed them on Genetic Analyser Model 3100 and 3700 (Applied Biosystems) according to the manufacturer's protocol. Sequencing was conducted from both ends using the same primers in the *trn*L–*rpl*32F spacer as in PCR. In this study, because we could not sequence the total range of *trn*L–*rpl*32F which was too long, we tried to sequence the region from each side as long as possible. Thus, we separately showed the sequence as two regions from each end in *trn*L–*rpl*32F. We designed several internal sequencing primers in the *trn*S(GCU)–*trn*G(UCC) intergenic region to read from one direction: *trn*S2: 5′-AGTCCCCTCAGCCATCTCTC-3′; *trn*G2: 5′-GTGTTGACACTTTGTCTCAC-3′; *trn*G3 5′-GTCCCTTGAACAAGTAAATGAG-3′. For all minor variants, we performed the sequencing at least twice to verify the variations.

### Phylogeographic analyses

All sequences were aligned using ClustalW (Thompson et al. 1994), and ambiguously aligned regions caused by indels were corrected manually. The cpDNA haplotypes were determined on the basis of these aligned sequences, and a haplotype network based on statistical parsimony was created to evaluate possible relationships between the haplotypes using TCS 1.06 (Clement et al. 2000). All indels were treated as point mutations and evenly weighted with other mutations. Haplotype diversity and within a population (*h*S), total species diversity (*h*T), and *G*ST values were computed according to the method of Nei (1987), which is based on haplotype frequencies. All calculations were performed with the program HAPLODIV (Pons and Petit 1995). Population relationships were determined using neighbor-joining (NJ) trees (Saitou and Nei 1987) obtained with POPTREE2 (Takezaki et al. 2010). The *D*A distance (Nei et al. 1983) was used to estimate divergence among haplotypes, and bootstrap support was estimated with 1000 replicates using POPTREE2 (Takezaki et al. 2010).

To determine the phylogeographic structure of *H. sieboldiana* and *H. albomarginata*, spatial analyses of molecular variance (SAMOVAs) were performed to identify groups of populations that are geographically homogeneous and maximally differentiated from each other using SAMOVA 1.0 (Dupanloup et al. 2002). This software simulates different partitions of *n* populations into *K* groups and retains the partition with the highest *F*CT value (i.e., the proportion of genetic variation among groups). Assuming that the final configuration was influenced by the initial configuration, 100 initial conditions were used as recommended by Dupanloup et al. (2002). Values of *K* in the range 2–10 were tested. Although *K* with the highest *F*CT represents the best number of groups and the best population configuration, it does not consistently represent a significant configuration. In particular, the final configuration of *K* with one or more single population group(s) could not derive the group structure (Heuertz et al. 2004). Thus, the number of groups (*K*) and the geographic structure were inferred from the configuration with the highest *F*CT that did not contain any single population group. We performed SAMOVAs for the data both with and without indels (Table 1).

**Table 1:** Information about sampling of populations in *Hosta sieboldiana* (a) and *Hosta albomarginata* (b)

Population code	Locality	Abbreviation	Latitude (N)	Longitude (E)	Haplotype (number of individuals)
(a) *Hosta sieboldiana*					
1	Atsuta, Hokkaido	Hokkaido1	43°22′	141°26′	A(10)
2	Inahopass, Hokkaido	Hokkaido2	43°01′	140°40′	A(10)
3	Hoshioki Waterfall, Hokkaido	Hokkaido3	43°07′	141°12′	A(9)
4	Shichinohe-machi, Aomori Pref.	Aomori	40°41′	141°08′	B(2), C(8)
5	Iwate-machi, Iwate Pref.	Iwate	39°58′	141°12′	B(3), C(6)
6	Dairakyo Gorge, Akita Pref.	Akita3	40°24′	140°21′	B(10)
7	Downstream of Dairakyo Gorge, Akita Pref.	Akita2	40°23′	140°18′	C(9)
8	Dakigaeri Valley, Akita Pref.	Akita1	39°36′	140°39′	C(7)
9	Tazawa Lake, Akita Pref.	Akita4	39°42′	140°42′	C(9)
10	Yuzawa, Morioka, Iwate Pref.	Morioka	39°39′	141°06′	C(10)
11	Ginzan Hotspring, Yamagata Pref.	Yamagata2	38°34′	140°31′	B(2), C(8)
12	Otaki Waterfall, Higashine, Yamagata Pref.	Yamagata	38°24′	140°30′	C(10)
13	Yone, Sakegawa-mura, Yamagata Pref.	Yamagata3	38°46′	140°12′	C(7)
14	Hachiman, Sendai, Miyagi Pref.	Miyagi1	38°16′	140°49′	C(10)
15	Hirose River, Miyagi Pref.	Miyagi2	38°16′	140°44′	B(5), C(2)
16	Wakigawa, Niigata Pref.	Niigata2	38°26′	139°28′	B(2)
17	Nametsuo Waterfall, Miyagi Pref.	Miyagi3	38°00′	140°23′	B(10)
18	Katagai, Sekikawa-mura, Niigata Pref.	Niigata3	38°04′	139°36′	B(9)
19	Ichinosawa, Toka-machi, Niigata Pref.	Niigata	37°10′	138°47′	C(9)
20	Uonuma, Niigata Pref.	Niigata4	37°12′	138°57′	C(1)
21	Ryujin Gorge, Ibaraki Pref.	Ibaraki1	36°58′	140°46′	C(9)
22	Mt. Tsukuba, Ibaraki Pref.	Ibaraki2	36°22′	140°10′	C(10)
23	Yamamoto, Itoigawa, Niigata Pref.	Niigata5	36°59′	137°52′	C(7)
24	Mt. Omine, Nagano Pref.	Nagano	36°31′	138°10′	C(5)
25	Obuchisawa, Yamanashi Pref.	Yamanashi	35°51′	138°19′	C(5)
26	Owa, Otaki-mura, Saitama Pref.	Saitama1	35°56′	138°57′	C(8)
27	Matsueda, Yokoze-mura, Saitama Pref.	Saitama2	35°56′	139°09′	C(5)
28	Komatsu, Ishikawa Pref.	Ishikawa	36°21′	136°30′	C(9)
29	Amoupass, Gifu Pref.	Gihu2	36°16′	136°56′	C(1)
30	Tokonigi Waterfall, Toyama Pref.	Toyama	36°28′	137°14′	C(7)
31	Osakapass, Takayama, Gifu Pref.	Gihu3	36°14′	137°14′	C(7)
32	Shirakawa, Gifu Pref.	Gihu	35°34′	137°11′	C(9)
33	Yogo, Shiga Pref.	Shiga	35°37′	136°14′	D(10)
34	Neba, Nagano Pref.	Nagano2	35°14′	137°37′	C(8)
35	Mt. Tadagatake, Fukui Pref.	Fukui	35°27′	135°45′	C(9)
36	Mt. Ryumon, Wakayama Pref.	Wakayama	34°16′	135°24′	C(6)
37	Kurumino River, Tottori Pref.	Dotori	35°22′	134°24′	C(10)
38	Mt. Shimo-hiruzen, Okayama Pref.	Okayama	35°19′	133°42′	C(3)
39	Yamano Gorge, Hukuyama, Hiroshima Pref.	Hiroshima	34°40′	133°21′	C(10)
40	Tachikue Gorge, Shimane Pref.	Shimane	35°17′	132°44′	C(7)
41	Chomon Gorge, Yamaguchi Pref.	Yamakuchi	34°31′	131°58′	C(10)
42	Shiozuka Plateau, Tokushima Pref.	Tokushima1	33°56′	133°40′	C(10)
43	Shiozuka Plateau, Tokushima Pref.	Tokushima2	33°55′	133°40′	C(10)
44	Mt. Aso, Kumamoto Pref.	Kumamoto	32°59′	130°58′	C(9)
45	Yakushima Is., Kagoshima Pref.	Yakushima	30°20′	130°30′	C(10)
(b) *Hosta albomarginata*					
1	Shotonbetsu, Hokkaido	Hokkaido1	44°49′	142°18′	a(10)
2	Onuma Park, Onuma-cho, Hokkaido	Hokkaido2	41°59′	140°40′	a(9)
3	Utasai, Kuromatsunai-cho, Hokkaido	Hokkaido3	42°37′	140°21′	a(8), b(1)
4	Bibai, Hokkaido	Hokkaido4	43°19′	141°51′	a(8)
5	Kiritappu, Hamanaka-cho, Hokkaido	Hokkaido5	43°05′	145°07′	a(9), b(1)
6	Erimo, Hokkaido	Hokkaido6	41°55′	143°14′	a(4), b(5)
7	Onbetsu, Hokkaido	Hokkaido7	42°56′	143°54′	a(9), b(1)
8	Imabetsu, Aomori Pref.	Aomori6	41°10′	140°28′	b(2)
9	Morita-cho, Aomori Pref.	Aomori1	40°78′	140°34′	b(8), c(1)
10	Bense Wetlands, Aomori Pref.	Aomori7	40°51′	140°17′	b(7)
11	Higashidori, Aomori Pref.	Aomori5	41°15′	141°18′	b(9)
12	Suwanosawa, Aomori Pref.	Aomori4	40°49′	140°50′	b(10)
13	Mt. Hakkoda, Aomori Pref.	Aomori2	40°41′	140°54′	b(5), c(5)
14	Hachinohe, Aomori Pref.	Aomori3	40°30′	141°33′	b(10)
15	Sashimaki Wetlands, Akita Pref.	Akita1	39°41′	140°41′	b(10)
16	Takayashiki, Akita Pref.	Akita4	39°31′	140°23′	b(8)
17	Nakajimachincho Pond, Akita Pref.	Akita3	39°19′	140°02′	c(2)
18	Mt. Chokai, Akita Pref.	Akita2	39°06′	140°02′	c(10)
19	Kesennuma, Miyagi Pref.	Miyagi1	38°49′	141°33′	b(9)
20	Yone, Sakewaga-mura, Yamagata Pref.	Yamagata3	38°46′	140°12′	b(5)
21	Funagawa-machi, Yamagata Pref.	Yamagata1	38°39′	140°15′	b(3)
22	Kunimigaoka, Sendai, Miyagi Pref.	Miyagi2	38°16′	140°49′	b(8), d(2)
23	Kuzuoka, Sendai, Miyagi Pref.	Miyagi3	38°17′	140°49′	b(10)
24	Kakuda, Miyagi Pref.	Miyagi4	37°59′	140°39′	b(10)
25	Marumori, Miyagi Pref.	Miyagi5	37°53′	141°05′	b(10)
26	Iide, Yamagata Pref.	Yamagata2	38°00′	139°56′	b(10)
27	Shichikashuku, Miyagi Pref.	Miyagi7	38°01′	140°18′	b(9)
28	Mt. Shirahagi, Miyagi Pref.	Miyagi6	38°07′	140°32′	b(10)
29	Nida Pond, Fukushima Pref.	Fukushima3	37°41′	140°18′	b(7)
30	Tainai, Niigata Pref.	Niigata2	38°02′	139°28′	b(7)
31	Gosen, Niigata Pref.	Niigata4	37°42′	139°13′	b(10)
32	Uonuma, Niigata Pref.	Niigata1	37°12′	138°57′	b(7)
33	Eryuda Waterfall, Fukushima Pref.	Fukushima1	36°58′	140°28′	b(6)
34	Samegawa, Fukushima Pref.	Fukushima2	36°56′	140°34′	b(6)
35	Mt. Tsukuba, Ibaraki Pref.	Ibaraki	36°22′	140°10′	b(10)
36	Himegawa, Niigata Pref.	Niigata3	36°37′	137°50′	b(8)
37	Miasa-mura, Nagano Pref.	Nagano	36°36′	137°53′	b(1), e(9)
38	Ueno, Tatsuno-machi, Nagano Pref.	Nagano3	36°00′	138°02′	e(10)
39	Obuchisawa, Yamanashi Pref.	Yamanashi	35°51′	138°19′	b(1), e(5)
40	Otaki, Saitama Pref.	Saitama2	35°57′	139°00′	b(7)
41	Matsueda, Yokoze-mura, Saitama Pref.	Saitama1	35°56′	139°09′	b(7)
42	Omachi, Nagano Pref.	Nagano2	36°32′	137°51′	b(7)
43	Komatsu, Ishikawa Pref.	Ishikawa	36°21′	136°30′	b(4), g(1)
44	Osakapass, Takayama, Gifu Pref.	Gihu3	36°14′	137°14′	b(10)
45	Hirugano Plateau, Gifu Pref.	Gihu2	36°00′	136°54′	b(7)
46	Tomika, Gifu Pref.	Gihu	35°30′	136°58′	b(10)
47	Ikenokouchi Wetlands, Fukui Pref.	Fukui	35°38′	136°03′	b(5)
48	Mt. Shimo-hiruzen, Okayama Pref.	Okayama	35°19′	133°42′	f(6)
49	Akana Wetlands, Shimane Pref.	Shinema	34°59′	132°42′	f(3), g(2)
50	Hiraodai Plateau, Kitakyushu, Fukuoka Pref.	Fukuoka	33°45′	130°53′	g(2)
51	Inosedo Wetlands, Oita Pref.	Oita	33°17′	131°25′	f(3), g(2)
52	Mt. Aso, Kumamoto Pref.	Kumamoto1	32°55′	131°11′	g(5)
53	Hitoyoshi, Kumamoto Pref.	Kumamoto2	32°11′	130°46′	h(10)
54	Okuchi, Kagoshima Pref.	Kagoshima	32°02′	130°36′	g(10)
55	Mt. Saragamine, Ehime Pref.	Ehime	33°49′	132°46′	b(10)

The geographic locations of genetic discontinuities among populations across the entire range covered by each species were assessed with Monmonier's maximum difference algorithm implemented in Barrier 2.2 (Manni et al. 2004). This program first creates a map of the sampling locations from geographic coordinates. From a matrix of genetic distances between populations, barriers are then represented on the map by identifying the edges of polygons where the maximum distances occur.

## Results

### Diversity of cpDNA sequences

**Figure 2:**
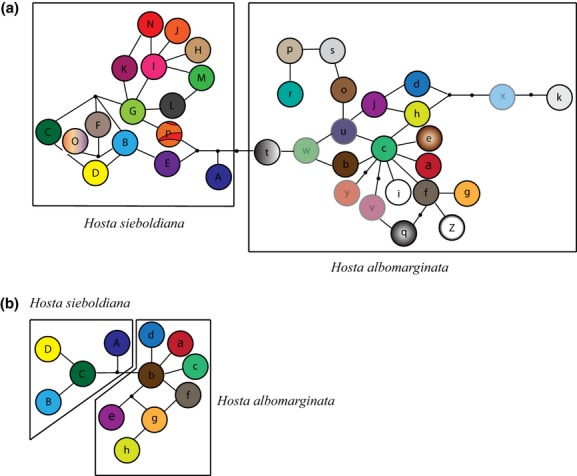
Statistical parsimony network of the cpDNA haplotypes of *Hosta sieboldiana* and *Hosta albomarginata* in the case of including indels (a) and excluding indels (b) for sequencing analysis data. Capital letter means the cpDNA haplotypes of *H*. *sieboldiana* and small letter means the cpDNA haplotypes of *H*. *albomarginata*.

**Figure 3:**
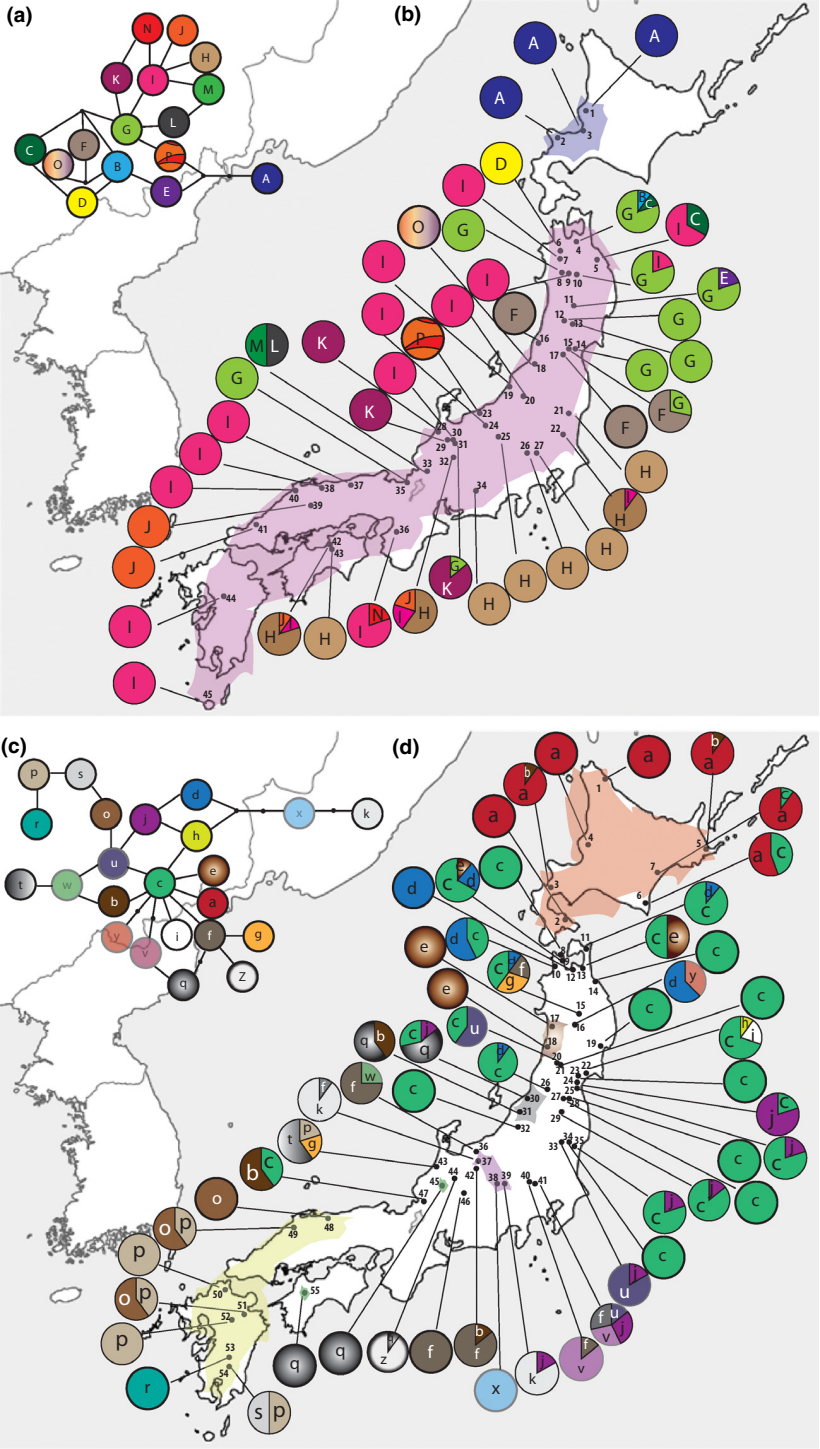
Statistical parsimony network of the cpDNA haplotypes of *Hosta sieboldiana* (a) and *Hosta albomarginata* (c) in the case of including indels for sequencing analysis data. Geographic distribution of the cpDNA of *H*. *sieboldiana* (b) and *H*. *albomarginata* (d). Two groups by SAMOVA are indicated using colors in *H*. *sieboldiana* and seven groups in *H*. *albomarginata*.

Based on approximately 1380 bp of the two noncoding regions of cpDNA, four substitutions, two indels, and eight types of *poly*T tructs (repeated sequences of T) were detected among sequences for *H. sieboldiana* (Table 2a). In contrast, seven substitutions, six indels, and 13 types of *poly*T tructs were found among sequences for *H. albomarginata* (Table 2b). All of the sequences obtained were deposited in DDBJ (accession Nos. AB787295–AB787447). When indels were included in the sequencing data analyses, 16 haplotypes were found in a total of 362 individuals from 45 populations of *H. sieboldiana*, whereas 23 haplotypes were found in a total of 436 individuals from 55 populations of *H. albomarginata* (Table 2a,b). We analyzed the two species using TCS network at first. However, we found that the two species have no common haplotypes probably because these species are not so much closely related (Fig. 2). So, we analyzed the two species separately. The genetic relationships between the haplotypes of *H. sieboldiana* and *H*. *albomarginata* were revealed in the parsimony network (Fig. 3a,c).

**Table 2:** (a) Polymorphic sites and cpDNA haplotypes based on sequences of two noncoding regions from *Hosta sieboldiana* (in the case of including the indels), (b) *Hosta albomarginata* (in the case of including the indels), (c) *Hosta sieboldiana* (in the case of excluding the indels), (d) *Hosta albomarginata* (in the case of excluding the indels)

Haplotype	*trnL*		*rpl32F*	*trnG – trnS*					
	
	128	244	244	213	325	617	720	781	853
(a)									
A	A	T12	T10	G	–	G	G	–	T11
B	C	T13	T10	T	–	G	T	–	T11
C	C	T13	T10	T	I1	G	T	–	T12
D	C	T13	T10	T	I1	G	T	–	T11
E	C	T12	T10	T	–	G	T	–	T11
F	C	T13	T10	T	–	G	T	I2	T12
G	C	T13	T10	G	–	G	T	–	T12
H	C	T15	T10	G	–	G	T	–	T12
I	C	T14	T10	G	–	G	T	–	T12
J	C	T14	T10	G	–	G	T	–	T11
K	C	T13	T10	G	–	G	T	–	T13
L	C	T13	T10	G	–	A	T	–	T12
M	C	T14	T10	G	–	A	T	–	T12
N	C	T14	T10	G	–	G	T	–	T12
O	C	T13	T11	T	–	G	T	I2	T11
P	C	T12	T10	G	–	G	T	–	T12

Haplotype	*trnL*			*rpl32F*			*trnG-trnS*									
		
	105	232	244	39	105	244	191	404	491	496	738	768	789	796	799	850
(b)																
a	T	C	T11	A	A	T11	–	–	T	C	T	–	–	–	–	T10
b	T	C	T12	A	A	T11	–	–	T	C	A	–	–	–	–	T10
c	T	C	T11	A	A	T11	–	–	T	C	A	–	–	–	–	T10
d	T	C	T10	A	A	T10	–	–	T	C	A	I3	–	–	–	T10
e	T	C	T11	A	G	T11	–	–	T	C	A	–	–	–	–	T10
f	T	C	T11	A	A	T12	–	–	T	C	A	–	–	–	–	T10
g	T	C	T11	A	A	T12	–	–	T	C	A	–	–	–	–	T11
h	T	C	T10	A	A	T11	–	–	T	C	A	–	–	–	–	T10
i	T	T	T11	A	A	T11	–	–	T	C	A	–	–	–	–	T10
j	T	C	T10	A	A	T10	–	–	T	C	A	–	–	–	–	T10
k	T	C	T10	A	A	T11	–	–	G	A	A	I3	I4	I5	–	T10
o	T	C	T11	T	A	T10	–	–	T	C	A	–	–	–	–	T10
p	T	C	T10	T	A	T10	–	–	G	C	A	–	–	–	–	T10
q	T	C	T11	A	A	T12	–	I2	T	C	A	–	–	–	–	T9
r	G	C	T10	T	A	T10	–	–	G	C	A	–	–	–	–	T10
s	T	C	T11	T	A	T10	–	–	G	C	A	–	–	–	–	T10
t	T	C	T12	A	A	T10	–	–	T	C	A	–	–	–	–	T11
u	T	C	T11	A	A	T10	–	–	T	C	A	–	–	–	–	T10
v	T	C	T11	A	A	T11	–	I2	T	C	A	–	–	–	–	T9
w	T	C	T12	A	A	T10	–	–	T	C	A	–	–	–	–	T10
x	T	C	T10	A	A	T11	–	–	G	A	A	I3	–	–	–	T10
y	T	C	T11	A	A	T11	–	–	T	C	A		I4	–	I6	T10
z	T	C	T11	A	A	T12	I1	I2	T	C	A	–	–	–	–	T9

Haplotype	*trnL*	*trnG – trnS*		

	128	213	617	720
(c)				
A	A	G	G	G
B	C	T	G	T
C	C	G	G	T
D	C	G	A	T

Haplotype	*trnL*		*rpl32F*		*trn*G-*trn*S		
		
	105	232	39	105	491	496	738
(d)							
a	T	C	A	A	T	C	T
b	T	C	A	A	T	C	A
c	T	C	A	G	T	C	A
d	T	T	A	A	T	C	A
e	T	C	A	A	G	A	A
f	T	C	T	A	T	C	A
g	T	C	T	A	G	C	A
h	G	C	T	A	G	C	A

(a) I1: inserted sequence = ATTATTTAATATCAATATATGTATGTGTTTGAT, I2: deleted sequence = TTAAAT.

Tnumber means “the number of tract.”

I1 and I2 mean the inserted sequence and the deleted sequence above, respectively.

(b) I1: deleted sequence = CTGCATTTCTTTTTTCGTTTTCAACTTCCCTTTTAACTGCCGGATTTTATGAATTTGAGTTCGGATTTAT, I2: deleted sequence = A, I3: deleted sequence = AATATAA, I4: inserted sequence = CGTTATT, I5: inserted sequence = ATT, I6: inserted sequence = TTATCCTTATAA Tnumber means “the number of track” I1, I2, and I3 mean the deleted sequence above I4, I2, and I6 mean the inserted sequence above.

For *H. sieboldiana*, only haplotype A was detected on Hokkaido, while haplotypes G, H, and I were dominant in the Japanese archipelago except for Hokkaido. Haplotype G was found in northern Japan, and haplotype H was indentified on the Pacific Sea side of the middle of Honshu and Shikoku. Haplotype I was detected on the Sea of Japan side of Honshu and Kyushu. The private haplotypes K and L were only found in the central region of Honshu (population 33; Fig. 3b).

For *H. albomarginata*, haplotype a was only detected on Hokkaido, while haplotype c was widespread throughout the middle and northern Honshu. Haplotypes o and p were detected in southwestern Honshu and Kyushu, while haplotypes s and r were found only in Kyushu. The private haplotypes k and x were found only in the central area of Honshu (populations 37–39; Fig. 3d).

**Figure 4:**
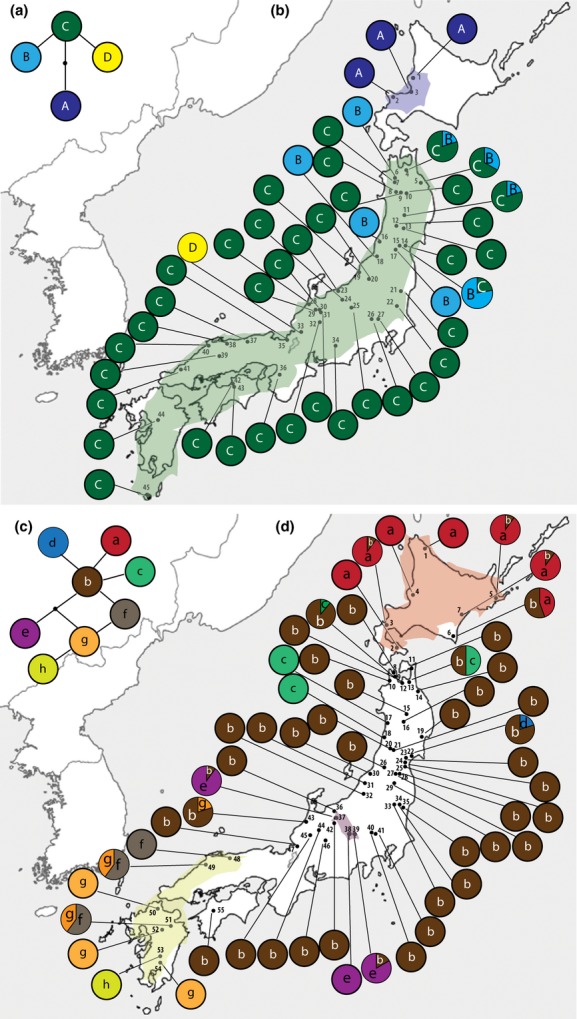
Statistical parsimony network of the cpDNA haplotypes of *Hosta sieboldiana* (a) and *Hosta albomarginata* (c) in the case of excluding indels for sequencing analysis data. Geographic distribution of the cpDNA of *H*. *sieboldiana* (b) and *H*. *albomarginata* (d). Two groups by SAMOVA are indicated using colors in *H*. *sieboldiana* and four groups in *H*. *albomarginata*.

When the sequence data were analyzed without indels, four haplotypes were revealed in *H. sieboldiana*, while eight haplotypes were detected in *H. albomarginata* (Table 2c,d). The genetic relationships between the haplotypes of *H. sieboldiana* and *H. albomarginata* are shown in the parsimony network (Fig. 4a,c). For *H. sieboldiana*, haplotype A was only detected on Hokkaido and haplotype C was dominant and widespread throughout the Japanese archipelago except on Hokkaido. The private haplotype D was found only in the central part of Honshu (population 33), as was the case when indels were included (Fig. 4b). For *H. albomarginata*, haplotype a was detected only on Hokkaido and haplotype b was dominant in the Japanese archipelago except for Kyushu, while haplotypes g and f were detected near southwestern Japan. The private haplotype e was only found in the central part of Honshu, as was the case when indels were included (populations 37–39; Fig. 4d).

### Geographic structure of genetic variation and haplotype diversity

The SAMOVA identified several phylogeographic groups in *H*. *sieboldiana* and *H*. *albomarginata*. When indels were included in the sequence analysis, the number of groups with the highest *F*CT that included no single population group was two (*K* = 2) for *H. sieboldiana* (Fig. 3b). The first group contained three populations with haplotype A on Hokkaido. The second group consisted of 42 populations in the Japanese archipelago except for Hokkaido.

In contrast, the number of groups with the highest *F*CT was seven (*K* = 7) for *H. albomarginata* (Fig. 3d). The first group consisted of six populations on Hokkaido except for the Erimo district in southeast Hokkaido (population 6). The second group consisted only of three populations in central Honshu (populations 37, 38 and 39). The third group consisted of seven populations in southwestern Japan and Kyushu (populations 48–54). The fourth group consisted of two populations of the Sea of Japan in north–central Honshu (populations 17 and 18). The fifth group consisted of two populations of north–central Honshu (populations 30 and 31). The sixth group consisted of two populations in relatively remote areas (populations 45 and 55), and the seventh group contained the remaining 35 populations in the southeast district of Hokkaido and Honshu.

When indels were excluded from the sequence analysis, the number of groups was two in *H. sieboldiana* and the groups determined by the SAMOVA were the same when indels were included (Fig. 4b). However, the number of groups was four in *H. albomarginata* (Fig. 4d). Furthermore, three groups were detected both with and without indels included in the sequence analysis.

Three additional groups were detected for *H*. *albomarginata* when indels were included compared with when indels were excluded. One group in northern Honshu (populations 17 and 18) included haplotype e when indels were included, and this haplotype corresponded to haplotype c, which occupied narrow regions when indels were excluded. When indels were excluded, haplotype c showed one mutation difference from haplotype b in the four groups identified by the SAMOVA, which is widely distributed in Honshu. One group in the north–central part of Honshu (populations 30 and 31) included haplotypes b, c, j, and q when indels were included, and when indels were excluded, these haplotypes all matched haplotype b, which dominates Honshu. These haplotypes exhibited different repeat numbers in a *poly*T region in the sequencing data. Thus, when indels were excluded, haplotypes b, c, j, and q corresponded to only haplotype b in the four groups identified by the SAMOVA. One group in the relatively remote areas (populations 45 and 55) contained haplotype q when indels were included, and this haplotype corresponded to haplotype b in the four groups identified by SAMOVA. Consequently, if we exclude indels from the sequence analysis, many haplotypes are assigned to specific haplotypes, such as haplotype b, which was common in the phylogeographic structures. As a result of the analysis of cpDNA noncoding regions in *H. sieboldiana* and *H. albomarginata*, we found only a few substitutions. We thought that it is difficult to elucidate the phylogeographic history of two species with only a few substitutions. Therefore, we tried to include all the information for the analysis of phylogeographic study as possible as we can. However, regardless of including indels or excluding indels in our analysis, we did not find large differences.

**Figure 5:**
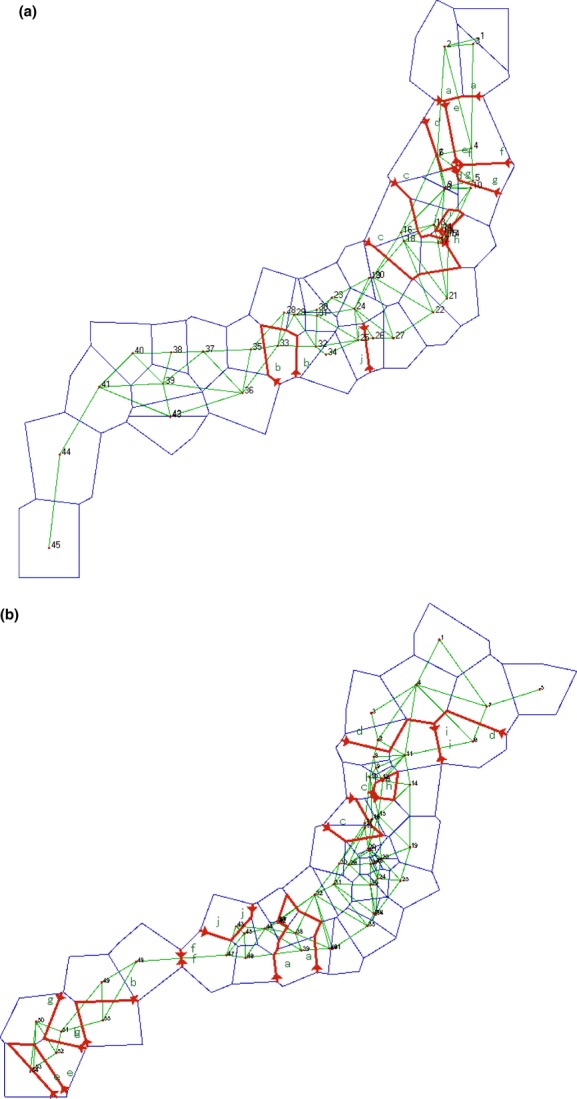
Geographic breaks identified in *Hosta sieboldiana* (a) and *Hosta albomarginata* (b) in the Japanese archipelago using Monmonier's algorithm. The current geographic distribution of each species is outlined in red, with breaks as recovered in the software program BARRIER 2.2 (Manni et al. 2004).

We revealed geographic genetic boundaries by Monmonier's algorithm. Boundaries were much more divided in both species than the SAMOVA analysis, and we found the similar patterns in the BARRIER analysis as in the SAMOVA analysis. In *H*. *sieboldiana*, we detected the boundaries between Hokkaido populations and the other regions at the first boundary (Fig. 5a). However, we found more boundaries in Honshu. One of populations in the central region of Honshu (Population 33) showed the independent boundary. The several populations in the north part of Honshu (populations 16, 17, and 18) were surrounded by the same barriers. In *H*. *albomarginata*, we also found the same boundaries as in the SAMOVA analysis. Four groups detected by the SAMOVA were also separated by the BARRIER (Fig. 5b).

Total genetic haplotype diversity (*h*T = 0.352 and 0.529 for *H*. *sieboldiana* and *H*. *albomarginata*, respectively) based on cpDNA data across all populations was much higher than the average within-population haplotype diversity (*h*S; 0.040 and 0.085, respectively) in both species (Table 3a,b). Given that total genetic SAMOVA group diversity based on SAMOVA group data, *H. albomarginata* (*h*T = 0.473) has higher values than *H. sieboldiana* (*h*T = 0.136), and given the average within-population SAMOVA group diversity (*h*S) based on SAMOVA group data, the value was 0 in the two species (Table 3c,d). The average within-population haplotype diversities and the total genetic haplotype diversity in *H. sieboldiana* and *H*. *albomarginata* showed conspicuous differences. However, the population differentiation (*G*ST) of *H*. *sieboldiana* and *H*. *albomarginata* showed almost the same values (0.839 and 0.886, respectively; Table 3a,b). This suggests that *H*. *albomarginata* exhibited greater genetic diversity at both the species and the population levels than *H*. *sieboldiana*.

**Table 3:** Comparison of several method of analysis of diversity of cpDNA and SAMOVA groups in (a,c) *Hosta sieboldiana* and (b,d) *Hosta albomarginata*. The method used is given in brackets: (*bias*), uncorrected definitions of the parameter; (*Nei*), estimates of Nei and Chesser (1983); (*Neib*), alternative estimates of Nei (1986); (*WC*), estimates of Weir and Cockerham (1984); (*PP*), Pons and Petit (1995)

Haplotypes	A	B	C	D	Total
(a)					
Frequency	0.07143	0.10590	0.79887	0.02381	1.00000
*h*S(*bias*)	0.00000	0.03554	0.03554	0.00000	0.03554
*h*S(*Nei*)	0.00000	0.04064	0.04064	0.00000	0.04064
*h*S(*WC*)	0.00000	0.04191	0.04191	0.00000	0.04191
*h*S(*PP*)	0.00000	0.04017	0.04017	0.00000	0.04017
SD	0.00000	0.01960	0.01960	0.00000	0.01960
*h*T(*bias*)	0.13265	0.18936	0.32136	0.04649	0.34493
*h*T(*Nei*)	0.13265	0.18949	0.32148	0.04649	0.34505
*h*T(*WC*)	0.07626	0.10341	0.17729	0.02781	0.38477
*h*T(*PP*)	0.13589	0.19312	0.32833	0.04762	0.35248
SD	0.06895	0.06828	0.07058	0.04535	0.08482
*G*ST(*bias*)	1.00000	0.81233	0.88941	1.00000	0.89697
*G*ST(*Nei*)	1.00000	0.78553	0.87359	1.00000	0.88222
*G*ST(*Neib*)	1.00000	0.78956	0.87622	1.00000	0.88470
*θ*(*WC*)	1.00000	0.79736	0.88180	1.00000	0.89108
*G*ST(*PP*)	1.00000	0.79179	0.87764	1.00000	0.88602
SD	–	–	0.05868	–	0.05842

Haplotypes	A	b	c	d	e	f	g	h	Total
(b)									
Frequency	0.11795	0.67543	0.03098	0.00385	0.05256	0.04231	0.05769	0.01923	1.00000
*h*S(*bias*)	0.02022	0.05474	0.01341	0.00615	0.00880	0.01846	0.02462	0.00000	0.07321
*h*S(*Nei*)	0.02326	0.06297	0.01543	0.00708	0.01013	0.02124	0.02832	0.00000	0.08421
*h*S(*WC*)	0.02596	0.06571	0.01791	0.00846	0.00916	0.01268	0.01691	0.00000	0.07840
*h*S(*PP*)	0.02265	0.06239	0.01496	0.00684	0.01026	0.02308	0.03077	0.00000	0.08547
SD	0.01245	0.01972	0.01143	0.00684	0.00741	0.01616	0.01770	0.00000	0.02436
*h*T(*bias*)	0.20807	0.43845	0.06005	0.00766	0.09960	0.08104	0.10873	0.03772	0.52066
*h*T(*Nei*)	0.20813	0.43861	0.06008	0.00768	0.09963	0.08109	0.10880	0.03772	0.52087
*h*T(*WC*)	0.11700	0.22410	0.03636	0.00464	0.05365	0.02754	0.04506	0.02316	0.53153
*h*T(*PP*)	0.21176	0.44597	0.06096	0.00769	0.10138	0.08226	0.11038	0.03846	0.52943
SD	0.06558	0.04304	0.04011	0.00763	0.05340	0.04535	0.05081	0.03698	0.07423
*G*ST(*bias*)	0.90283	0.87514	0.77660	0.19691	0.91161	0.77218	0.77361	1.00000	0.85940
*G*ST(*Nei*)	0.88825	0.85642	0.74319	0.07832	0.89835	0.73810	0.73974	1.00000	0.83833
*G*ST(*Neib*)	0.89017	0.85880	0.74687	0.07973	0.90011	0.74184	0.74346	1.00000	0.84094
*θ*(*WC*)	0.88908	0.85339	0.75372	0.08929	0.91462	0.76974	0.81234	1.00000	0.85250
*G*ST(*PP*)	0.89304	0.86010	0.75464	0.11111	0.89883	0.71947	0.72124	1.00000	0.83856
SD	0.03822	0.03620	–	–	0.03435	–	–	–	0.04282

SAMOVA group	Group1	Group2	Total
(c)			
Frequency	0.07143	0.92875	1.00000
*h*S(*bias*)	0.00000	0.00000	0.00000
*h*S(*Nei*)	0.00000	0.00000	0.00000
*h*S(*WC*)	0.00000	0.00000	0.00000
*h*S(*PP*)	0.00000	0.00000	0.00000
SD	0.00000	0.00000	0.00000
*h*T(*bias*)	0.13265	0.13265	0.13265
*h*T(*Nei*)	0.13265	0.13265	0.13265
*h*T(*WC*)	0.07626	0.07626	0.15252
*h*T(*PP*)	0.13589	0.13589	0.13589
SD	0.06895	0.06895	0.06895
*G*ST(*bias*)	1.00000	1.00000	1.00000
*G*ST(*Nei*)	1.00000	1.00000	1.00000
*G*ST(*Neib*)	1.00000	1.00000	1.00000
*θ*(*WC*)	1.00000	1.00000	1.00000
*G*ST(*PP*)	1.00000	1.00000	1.00000
SD	–	–	–

SAMOVA group	Group1	Group2	Group3	Group4	Total
(d)					
Frequency	0.11538	0.05769	0.11538	0.71154	1.00000
*h*S(*bias*)	0.00000	0.00000	0.00000	0.00000	0.00000
*h*S(*Nei*)	0.00000	0.00000	0.00000	0.00000	0.00000
*h*S(*WC*)	0.00000	0.00000	0.00000	0.00000	0.00000
*h*S(*PP*)	0.00000	0.00000	0.00000	0.00000	0.00000
SD	0.00000	0.00000	0.00000	0.00000	0.00000
*h*T(*bias*)	0.20414	0.10873	0.20414	0.41050	0.46376
*h*T(*Nei*)	0.20414	0.10873	0.20414	0.41050	0.46376
*h*T(*WC*)	0.11549	0.05792	0.08795	0.20823	0.46959
*h*T(*PP*)	0.20814	0.11086	0.20814	0.41855	0.47285
SD	0.06883	0.05776	0.06883	0.05368	0.07718
*G*ST(*bias*)	1.00000	1.00000	1.00000	1.00000	1.00000
*G*ST(*Nei*)	1.00000	1.00000	1.00000	1.00000	1.00000
*G*ST(*Neib*)	1.00000	1.00000	1.00000	1.00000	1.00000
*θ*(*WC*)	1.00000	1.00000	1.00000	1.00000	1.00000
*G*ST(*PP*)	1.00000	1.00000	1.00000	1.00000	1.00000
SD	–	–	–	–	–

SD, standard deviates of the estimates (*PP*).

### Genetic structure among populations

**Figure 6:**
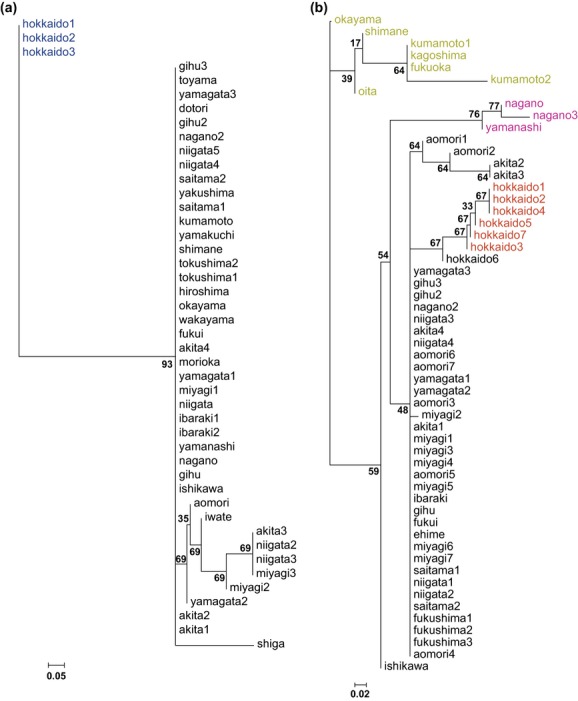
The neighbor-joining (NJ) tree based on chloroplast DNA (cpDNA) variations of *Hosta sieboldiana* (a) and *Hosta albomarginata* (b). The numbers above or below branches are bootstrap values (1000 replicates). Different colors mean different groups in the SAMOVA results.

To clarify the genetic relationship between the populations examined, phylogenetic trees were constructed using NJ methods (Fig. 6). The topology of the NJ tree based on the *D*A distance among the 45 populations of *H. sieboldiana* generally reflected their geographic locations (Fig. 6a) and showed one well-supported clade including three Hokkaido populations supported by the highest bootstrap value (93%). The populations including haplotype B were clustered with a 69% bootstrap value. For *H. albomarginata*, the topology of the NJ tree among the populations also reflected their geographic locations (Fig. 6b). Populations in Kyushu and southwestern Honshu (populations 48–54) were clustered by a 59% bootstrap value, and populations in the central region of Honshu (37, 38, and 39) were clustered by a 76% bootstrap value, while six populations (except population 6) on Hokkaido were clustered by a 67% bootstrap value.

## Discussion

### Geographic structure of *H. sieboldiana* in the Japanese archipelago

Regardless of whether indels were included, the results of the SAMOVA revealed two phylogeographic groups for *H. sieboldiana* in (1) Hokkaido and (2) Honshu, Shikoku and Kyushu. The BARRIER results also supported the SAMOVA results. According to previous phylogeographic studies, some plants in the Japanese archipelago show phylogeographic differentiation between Honshu and Hokkaido (Aizawa et al. 2009; Hu et al. 2010; Ohsawa et al. 2011). Hokkaido was separated from Honshu by the formation of the Tsugaru Strait 100,000–150,000 years ago. Thus, the Tsugaru Strait may have played a role in limiting successful migration during postglacial recolonization, and/or in restricting gene flow between established populations in Hokkaido and Honshu, and thus promoted genetic differentiation between them. The Tsugaru Strait has been a physical barrier to migration and gene flow in various animal and plant species. The populations of *H. sieboldiana* on Hokkaido have a narrow distribution range. These small population sizes may lead to a secondary loss of genetic variation as a result of bottleneck and genetic drift. The simple haplotype composition of *H. sieboldiana* on Hokkaido might be due to founder effects after migration across the sea from Honshu before the last glaciations. Okaura et al. (2007) suggested that the current Hokkaido populations of *Quercus mongolica* var. *crispula* and *Q*. *serrata* were established by expansion from Honshu following the LGM. Moreover, Ohsawa et al. (2011) also suggested that the northeastern group of *Quercus crispula* moved north from Honshu. The low diversity of haplotypes on Hokkaido suggests that *H*. *sieboldiana* was derived from refugia in Honshu to Hokkaido through the Tsugaru Strait. However, there is a possibility that the current populations on Hokkaido might have originated from the recolonization of the former refugia on Hokkaido and underwent a bottleneck and a genetic drift at and after the recolonization.

Group (2) covers most of the distribution area of *H. sieboldiana* in the Japanese archipelago except for Hokkaido. A part of Honshu may have had at least one refugium in response to the Quaternary climate changes although we could not specify the area. As the climate warmed during the postglacial period, *H*. *sieboldiana* rapidly expanded from refugia to northeastern and/or southwestern Japan. Furthermore, the population in central Honshu (population 33) in Group 2 included private haplotypes. Iwasaki et al. (2012) hypothesized the migration history of Japanese deciduous broad-leaved forests. They postulated that six separate refugia occurred during the LGM, after which the forests separately expanded from each of the refugia as the climate warmed. The private haplotypes found in the population 33 may be evidence of a refugium near the population. Iwasaki et al. (2012) indicated the existence of one of six refugia around this area.

### Geographic structure of *H. albomarginata* in the Japanese archipelago

When indels were included, the results of SAMOVA revealed seven phylogeographic groups in *H*. *albomarginata*. Conversely, when indels were excluded from the sequence data, the SAMOVA revealed four phylogeographic groups. Regardless whether indels were included, the SAMOVA identified three common groups. The BARRIER analysis also supports the SAMOVA results. The range of the first group comprised most of the populations on Hokkaido except for population 6, the southeast population. Similar to *H*. *sieboldiana*, *H. albomarginata* may have been derived from a refugium in Honshu to Hokkaido through the Tsugaru Strait, although unlike *H. sieboldiana*, *H. albomarginata* is widely distributed on Hokkaido. Thus, *H. albomarginata* might exhibit more diverse genetic structuring than *H*. *sieboldiana*.

The second group occupied only the central part of Honshu and harbored private haplotypes. Although attributing this finding to a single factor is difficult, one explanation for the private haplotype observed in this group is that these areas experienced bottleneck events. Aizawa et al. (2008) studied *Picea alcoquiana* and postulated that bottleneck events could have occurred around these areas and that the peripheral populations isolated by mountains experienced a recent bottleneck, thus reducing genetic diversity. The populations of this group might have also experienced the same range shifts as *P. alcoquiana* in response to the Quaternary climate changes.

The third group consists of Kyushu and southwestern Honshu populations. Previous phylogeographic studies revealed the same patterns (Sugahara et al. 2011). Sugahara et al. (2011) postulated that the woody species *Aesculus turbinata* in Japan might have consistently retained sufficiently large distribution ranges to harbor several haplotypes during the Quaternary climatic oscillations, causing the southwestern populations of *A. turbinata* to exhibit local phylogeographic structures or unique haplotypes. *Hosta albomarginata* might have experienced a similar range distribution history as *A. turbinate*, and some parts of these areas might have been a refugium of *H*. *albomarginata* during the Quaternary climate oscillations. Given the SAMOVA results and the BARRIER results, we hypothesized that a refugium existed within northern Honshu. The wide distribution of haplotype b might have been generated over a longer period prior to the LGM, which would explain the present haplotype distribution pattern. The population in the southeast district on Hokkaido (population 6) of the Honshu group differs from others on Hokkaido. Previous studies of *Veratrum album* ssp. *oxysepalum* showed the same phylogeographic pattern (Kikuchi et al. 2010). Kikuchi et al. (2010) suggested that more than three refugia (northern, central, and southern refugia) would have existed during the last glaciation and that populations in the central refugium would have expanded to the southeast area of Hokkaido via long-distance dispersal after the LGM. Similar to *Veratrum album* ssp. *oxysepalum*, *H. albomarginata* populations of the central refugium may have spread widely throughout northern Honshu and the southernmost part of Hokkaido.

If the three distinct distributions of cpDNA haplotypes that we identified originated in the glacial era, *H*. *albomarginata* may have migrated to the Japanese archipelago from the Eurasian continent via a landbridge from the Sakhalin and expanded throughout the Japanese archipelago before the last glaciation. This would mean that more than two refugia existed during the last glaciation: One in the south part of the Japanese archipelago and the other on Hokkaido. To test this hypothesis, one must include Russian populations in the analyses; however, such samples are not currently available. Czerepanov (1995) reported that *H*. *albomarginata* and *H*. *rectifolia* are well-differentiated species based on morphological features and habitats in Russia. *Hosta albomarginata* occurs only south of the Amurskaya and Primorsky Territories of the Eurasian continent, while *H*. *rectifolia* is distributed in Sakhalin and the Kurils (Gage et al. 2006). Maekawa (1940) also regarded populations of *H*. *albomarginata* in the northern parts of Honshu and Hokkaido as *H*. *rectifolia*. The extent of the differences between the genetic structures of *H*. *rectifolia* and *H*. *albomarginata* in Russia is not currently known. An analysis of this genetic differentiation would provide a more detailed understanding of the phylogeographic patterns of *H*. *albomarginata*.

### Comparison of *H. sieboldiana* and *H. albomarginata*

We analyzed the genetic structures of *H*. *sieboldiana* and *H*. *albomarginata*. The SAMOVA identified two groups in *H. sieboldiana* and four in *H. albomarginata*, and *H. sieboldiana* exhibited significantly lower haplotype diversity than *H. albomarginata*. The lower haplotype diversity in *H. sieboldiana* may have resulted from its relatively restricted habitat and geographic distribution. *Hosta sieboldiana* mostly occurs on cliffs around waterfalls and steep clines, thus restricting the interpopulation gene flow and leading to a smaller effective population size. Conversely, *H. albomarginata* widely inhabits wetlands, where regional exchanges among gene pools are more likely, leading to a relatively large effective population size. At a continental scale, *H*. *sieboldiana* is native to the Japanese archipelago, whereas *H. albomarginata* is distributed outside of the Japanese archipelago within the Eurasian continent, Sakhalin, and the Kurils of Russia. The relatively restricted geographic range of *H*. *sieboldiana*, combined with the relatively narrow distribution of appropriate habitat within its range, would have exacerbated the restrictions to gene exchanges after the LGM. In contrast, the larger distribution range of *H*. *albomarginata* might have enabled the maintenance of a larger gene pool.

Even though these two species exhibit different phylogeographic structures, we determined that they show the same distinct genetic structure on Hokkaido. Therefore, these species might have experienced the same historical distribution range across the Tsugaru Strait during the LGM (Aizawa et al. 2007; Hu et al. 2010; Ohsawa et al. 2011).

Hybridization has been suggested to provide the adaptive genetic variation needed to invade postglacial habitats (Stebbins 1972; Excoffier et al. 2009; Saeki et al. 2011). Although we currently have no evidence for hybridization between *H*. *sieboldiana* and *H*. *albomarginata*, the possibility exists that these species have hybridized (Takahashi 2002). Therefore, further studies analyzing nuclear DNA are required to examine hybridization between *H*. *sieboldiana* and *H*. *albomarginata*.

## Conclusions

*Hosta sieboldiana* and *H. albomarginata* may have experienced different distributional range shifts during the last glaciation. Similar conclusions were reported by previous studies (Maliouchenko et al. 2007; Toyama and Yahara 2009; Saeki et al. 2011). Our study also provides further evidence that different responses of migration routes and refugia to climate changes during the last glacial period may have been due to ecological differences, even between congeneric species with similar morphologies and life forms. Also, more detailed phylogeographic studies including *H. albomarginata* samples from Russian populations are necessary to test the validity of the comparative phylogeographic histories of the two species.
